# Supplementary material for: A handheld fiber-optic tissue sensing device for spine surgery
Source: PLoS One. 2024 Dec 19;19(12):e0314706. doi: 10.1371/journal.pone.0314706 (PMC11658522; doi:10.1371/journal.pone.0314706)
Supplement: Attachment — Submitted filename: Review comments.docx [file pone.0314706.s001.docx]

The manuscript entitled “A handheld fiber-optic tissue sensing device for spine surgery” has an interesting subject and useful results and it can be published after some revision according to the below comments:

- Please improve the English language and structure
- Article paragraphing should be improved
- Several explanatory schematics corresponding to the involved mechanisms should be added
- What are the key advantages of using a handheld fiber-optic tissue sensing device compared to traditional methods for pedicle screw placement in spine surgery?
- How does Diffuse Reflectance Spectroscopy (DRS) contribute to the differentiation of bone tissues during surgical procedures?
- What specific challenges were encountered in the development of the handheld fiber-optic device, particularly regarding coupling efficiencies?
- How does the integration of laser diodes at two distinct wavelengths enhance the functionality of the tissue-sensing device?
- What role does the printed circuit board (PCB) play in the operation of the handheld fiber-optic tissue sensing device?
- How does the microcontroller process the signals from the photodiode to provide real-time feedback to surgeons?
- What are the implications of using low-cost and readily available components in the design of the fiber-optic sensing device for widespread clinical adoption?
- In what ways does real-time audio feedback improve surgical accuracy and outcomes during spinal fusion procedures?
- What are the limitations of the prototype device, and how might future iterations address these issues?
- How does the device's compact design influence its usability in various healthcare settings?
- What potential improvements could be made to the signal amplification process to enhance the accuracy of tissue differentiation?
- How does the device ensure that the feedback provided to surgeons is both timely and reliable during surgery?
- The literature should be improved using the below papers
- Wang, Y., Zhai, W., Zhang, H., Cheng, S., & Li, J. (2023). Injectable Polyzwitterionic Lubricant for Complete Prevention of Cardiac Adhesion. Macromolecular Bioscience, 2200554. doi: https://doi.org/10.1002/mabi.202200554
- Yao, R., Ge, Z., Wang, D., Shang, N., & Shi, J. (2024). Self-sensing joints for in-situ structural health monitoring of composite pipes: A piezoresistive behavior-based method. Engineering Structures, 308, 118049. doi: https://doi.org/10.1016/j.engstruct.2024.118049
- Fu, Y., Chen, X., Song, W., Kuang, J., Wu, W., Yang, X.,... Liao, Y. (2024). Light-Switch Electrochemiluminescence-Driven microfluidic sensor for rapid and sensitive detection of Mpox virus. Chemical Engineering Journal, 498, 154930. doi: https://doi.org/10.1016/j.cej.2024.154930
- Zhou, Y., Xie, J., Zhang, X., Wu, W., & Kwong, S. (2024). Energy-Efficient and Interpretable Multisensor Human Activity Recognition via Deep Fused Lasso Net. IEEE Transactions on Emerging Topics in Computational Intelligence, 1-13. doi: 10.1109/TETCI.2024.3430008
- Jiang, Z., Han, X., Zhao, C., Wang, S., & Tang, X. (2022). Recent Advance in Biological Responsive Nanomaterials for Biosensing and Molecular Imaging Application. International Journal of Molecular Sciences , 23(3), 1923. doi: https://doi.org/10.3390/ijms23031923
- What are the broader implications of integrating tissue sensing technology into surgical instruments for other types of surgeries beyond spinal procedures?
- How does the study address the need for improved surgical accuracy in the context of rising demand for interventions related to age-related degenerative diseases?
- What future research directions could be pursued to further develop and optimize fiber-optic sensing technologies in surgical applications?
