# Supplementary material for: A handheld fiber-optic tissue sensing device for spine surgery
Source: PLoS One. 2024 Dec 19;19(12):e0314706. doi: 10.1371/journal.pone.0314706 (PMC11658522; doi:10.1371/journal.pone.0314706)
Supplement: Attachment — Submitted filename: rebuttal.docx [file pone.0314706.s002.docx]

Dear editor,

Thank you for reviewing our manuscript and providing us with valuable comments. We have revised the article to address all reviewers’ comments, and the suggested alterations are incorporated in the revised manuscript (highlighted in blue). Below, we respond to these comments in a point-wise fashion, where our responses are in *italic text*.

**Reviewer #1:**

Dear Authors,
The manuscript "A handheld fiber-optic tissue sensing device for spine surgery" presents a novel device designed to assist in real-time differentiation of bone tissue during spinal fusion surgery. The device uses DRS to aid surgeons in accurately placing pedicle screws, which is crucial for the success of spinal fusion procedures. The handheld prototype employs laser diodes at two wavelengths (940 nm and 1310 nm) to illuminate the tissue, with a photodiode detecting the reflected light, providing real-time feedback through audio signals.
The design emphasizes compactness, cost-effectiveness, and real-time feedback in surgical settings. Despite challenges related to fiber-optic coupling, the proof-of-concept demonstrated successful differentiation between cancellous and cortical bone tissues. The device shows potential to improve surgical accuracy and patient outcomes, though further testing and validation, including mechanical stress tests and clinical trials, are recommended.

Below are my comments:
The manuscript describes using a handheld device for real-time bone tissue differentiation. Could the authors provide more details on the selection criteria for the wavelengths used (940 nm and 1310 nm)? Why were these wavelengths prioritized, particularly considering the broader NIR spectrum?

*We thank the reviewer for their appreciation of our work and thoughtful feedback. The wavelengths for our prototype were carefully selected based on optimal wavelengths for distinguishing bone tissues identified in the literature [14, 19, 23-27]. As we were working with off-the-shelf laser diodes, the available wavelengths with appropriate power levels were limited to 690 nm, 905 nm, 940 nm, and 1270 nm. We chose 1310 nm as an additional reference wavelength (ll. 81-85). We did not extend our wavelength selection further into the broader NIR spectrum, as water absorption dominates at wavelengths beyond 1310 nm, making it difficult to distinguish cancellous and cortical bone. Through Monte Carlo simulations, we analyzed the expected signal difference between cancellous and cortical bone across these wavelengths and ultimately selected 940 nm and 1310 nm, as they provided the largest intensity differences (ll. 86-88). We have clarified this reasoning in the manuscript by adding: “While DRS typically employs a broadband light source for tissue illumination, we opted for two wavelengths specifically selected to allow the distinction between cancellous and cortical bone based on the ratio of their signals, avoiding wavelengths dominated by hemoglobin or water absorption, which could hinder tissue differentiation.” (ll. 67-72)*

The paper discusses challenges related to optical coupling efficiencies. Could the authors elaborate on how the device was calibrated across different tissue types? Was any baseline established for cancellous and cortical bones prior to clinical testing?

*Thank you for this important question. As our work primarily focuses on the technical development of a novel design, we were unable to conduct clinical testing during this phase. Instead, we focused on validating the device’s optical performance through Monte Carlo simulations and by testing it on a white reference standard and tissue-mimicking phantom. These tests allowed us to calibrate the device and establish a threshold ratio for distinguishing between cancellous and cortical bone. The final threshold will be determined on ex-vivo human tissue. We have clarified this in our discussion: “The reflection ratio Q for both cancellous and cortical bone should be computed for each reading and analyzed to verify the set threshold ratio and adjust it if necessary. The final threshold will be determined based on the results from experiments using human tissues. If Q_t_ successfully separates the distributions of Q_canc_ and Q_cort_, it indicates that the two tissue types can be distinguished. Otherwise, a more complex metric than the reflection ratio may be necessary.” (ll. 526-531)*

The device aims to improve pedicle screw placement accuracy during spinal fusion. How was it ensured that the device's real-time feedback remains reliable and consistent over extended periods?

*We ensured consistent light emission by controlling the laser output power, which was validated experimentally (ll. 361-366). This regulation guarantees reliable feedback throughout an entire spinal fusion procedure. We have updated the manuscript to reflect this: “Applying this current to pin AMD on the IC decreased the operating current and reduced the optical output power, confirming the driver's ability to autonomously regulate current to ensure consistent light output for reliable feedback even over extended periods.” (ll. 363-366)*

The design prioritizes cost and size, but surgical instruments typically experience significant mechanical stress. Have the authors conducted any stress tests or fatigue analyses to ensure the device’s functionality during extended or repeated use?

*We acknowledge the importance of mechanical robustness and thank the reviewer for raising this point. Since this work presents a proof-of-concept, mechanical factors were not the primary focus at this stage. However, we recognize the need for future iterations to prioritize enhanced mechanical robustness, especially for in-vivo operations. We have included this consideration in the revised manuscript: “Moreover, future iterations of our device must prioritize enhanced mechanical robustness to accommodate in-vivo operations, where surgeons apply considerable forces to the bone [53]. Drawing from experience in earlier ex-vivo and in-vivo tests of DRS probes [18, 20], we focused on securely coupling the optical fibers in this design. Redesign efforts should ensure that the device withstands the substantial mechanical stresses exerted on the handle and probe shaft during surgical procedures.” (ll. 545-550)*

While the manuscript mentions plans for future clinical validation, it would benefit from discussing any preliminary testing or feedback from surgeons. Have any initial trials been conducted, and if so, what were the key insights or takeaways?

*We appreciate this suggestion. Although we have collaborated with surgeons throughout the development process to ensure that our device addresses clinical needs, no formal usability trials have been conducted yet. We have revised the manuscript to emphasize the importance of this collaboration: “In addition to mechanical robustness and sensing capabilities, usability in clinical practice is essential, and close collaboration with surgeons is encouraged to ensure the device meets clinical needs and integrates seamlessly into surgical workflows.” (ll. 551-553)*

The manuscript references similar devices like PediGuard, which uses EIS. Can the authors elaborate on the advantages of DRS over EIS? In what specific clinical scenarios would DRS outperform EIS regarding surgical guidance?

*Thank you for this insightful question. The PediGuard system, which relies on EIS, can only detect tissue in direct contact with its tip, meaning that a breach can only be detected once it has already begun. In contrast, DRS enables early breach detection. Additionally, the PediGuard is susceptible to interference from blood and tissue accumulation in the pilot hole, whereas DRS, particularly in the NIR spectrum, is unaffected by blood interference. We have further emphasized these advantages in the revised manuscript: “While the PediGuard system (Spine-Guard SA, Vincennes, France) has pioneered tissue sensing in spine surgery using EIS, it faces challenges such as susceptibility to blood and tissue accumulation in the pilot hole [13, 14]. Additionally, as the device only measures tissue in direct contact with its tip, a cortical breach can only be identified once the breach is already in progress, limiting the opportunity for early intervention [13, 15].”* (ll. 25-31)

Since this device is intended for surgical use, could the authors expand on the sterilization process for the components? How will the integrity of the optical fibers and electronics be maintained after repeated sterilizations?

*We agree that sterilization and maintaining the integrity of the device's components post-sterilization are critical issues. The most straightforward solution would be to make the device single-use. However, a more sustainable approach could involve making the shaft and optical fibers disposable while reusing the remaining components. We have included this consideration and added sources on the sterilizability of optical fibers to our manuscript: “Achieving complete sterilizability poses challenges in ensuring the functionality of all components post-sterilization [54]. However, technologies for sterilizing fiber-based systems, such as fiber-optic endoscopes, are well-established [55], demonstrating that sterilization of optical components is feasible. Alternatively, a semi-reusable approach is imaginable, where only the shaft that comes in touch with the patient is disposable while the remaining components are reusable, balancing cost-effectiveness with environmental impact.” (ll. 556-561)*

**Reviewer #2:**

The manuscript entitled “A handheld fiber-optic tissue sensing device for spine surgery” has an interesting subject and useful results and it can be published after some revision according to the below comments:

• Please improve the English language and structure

*Thank you for your suggestion. We have carefully reviewed the entire manuscript for clarity and flow. We also performed a language check to further enhance the readability and ensure that the manuscript meets the high standards of academic writing.*

• Article paragraphing should be improved

*We appreciate the reviewer’s feedback. We have revisited the manuscript’s structure to ensure that the flow of information is logical and easy to follow.*

• Several explanatory schematics corresponding to the involved mechanisms should be added

*Thank you for the suggestion. We agree that clear visuals are essential for understanding the mechanisms. Our manuscript already includes explanatory figures for the electronics schematics of the dynamic driver circuit for the laser diodes as well as the two-stage TIA (Figures 2 and 3), which illustrate these key concepts and the operational mechanisms of the device. Additionally, we have provided a supplementary figure along with the uploaded data that includes the full schematics of the electronics circuit as implemented on the PCB.*

• What are the key advantages of using a handheld fiber-optic tissue sensing device compared to traditional methods for pedicle screw placement in spine surgery?

*Conventional methods for guiding pedicle screw placement, such as intraoperative fluoroscopy, computer-assisted navigation, and robotic assistance, offer significant accuracy improvements but are associated with high costs and the need for specialized training. These factors limit their widespread adoption, especially in resource-constrained settings. In contrast, our compact DRS-based device provides an affordable and portable alternative. It is built from low-cost, readily available components and delivers real-time feedback, without the need for large-scale systems or extensive operator training, making it accessible to a broader range of healthcare facilities. This advantage is reflected in our manuscript: “To enhance the accuracy of screw placement, various guidance systems such as intraoperative fluoroscopy, computer-assisted navigation, and robotic assistance have been developed [7]. While these technologies have shown great promise in improving accuracy, they come with drawbacks including high costs, the need for specialized training, and limitations in availability [8–10]. [...] Integrating [DRS] into surgical instruments presents a promising alternative for improving the accuracy of pedicle screw placement.” (ll. 12-23)*

• How does Diffuse Reflectance Spectroscopy (DRS) contribute to the differentiation of bone tissues during surgical procedures?

*We appreciate the reviewer’s question. DRS operates by illuminating tissue with light and measuring the light that is scattered and absorbed within the tissue. Light scattering is influenced by the structural characteristics of the tissue, while absorption is primarily determined by its biochemical composition. The pattern of diffuse reflectance that is captured at the tissue surface carries this information, which in turn allows for differentiation between cancellous and cortical bone. This is crucial for pedicle screw placement, where accurate identification of bone tissue is necessary. DRS is particularly effective in this regard because cortical and cancellous bone differ significantly in terms of composition, including fat content, which is a key factor detected by this technique. We have improved the introduction of our paper to clarify the mechanisms on which DRS is based: "DRS operates on the principle of light interaction with tissue to assess its composition. A spectrum of light is directed into tissue via optical fibers, inducing scattering and absorption processes that very according to the tissue's structure and biochemical composition. The light that is reflected back carries information about the tissue's optical properties.” (ll. 33-36)*

• What specific challenges were encountered in the development of the handheld fiber-optic device, particularly regarding coupling efficiencies?

*Thank you for raising this point. One of the key technical challenges we faced during development was achieving effective fiber coupling between the laser diodes and photodiode. Although we expected coupling efficiencies of approximately 30%, we encountered significantly lower actual values. Specifically, the coupling efficiency for the 940 nm laser diode was only 14%, while for the 1310 nm laser diode and the photodiode, it was 0%. This discrepancy is primarily due to difficulties in aligning the fiber and diode surfaces with high precision and ensuring proper optical adhesion. To improve this in future iterations, we recognize the need for reliable assembly tools and processes for butt-coupling to enhance coupling efficiency and optical performance. We have emphasized these challenges in the discussion section of our revised manuscript: “To mitigate these shortcomings, reliable assembly tools and processes for butt-coupling have to be established to align the fiber and diode surfaces with high precision.” (ll. 443-444)*

• How does the integration of laser diodes at two distinct wavelengths enhance the functionality of the tissue-sensing device?

*We appreciate the reviewer’s question regarding the role of the two laser diodes. The integration of laser diodes at two distinct wavelengths is not merely an enhancement but is crucial to the core functionality of the device. These two wavelengths are specifically selected to facilitate the application of DRS, allowing the device to differentiate between cancellous and cortical bone tissues. The mechanism of DRS, as described in the manuscript, relies on the interaction of light with tissue. The light scattered and absorbed by the tissue varies depending on its composition, and the reflected light carries this information back to the sensor. By selecting wavelengths where cortical and cancellous bone tissues show significant differences in optical properties, we can effectively distinguish between them using only two wavelengths. We have added further clarification to our manuscript to emphasize that these specific wavelengths were chosen to optimize bone tissue differentiation: “While DRS typically employs a broadband light source for tissue illumination, we opted for just two wavelengths. By specifically selecting wavelengths where cancellous and cortical bone tissues show significant differences in optical properties, we can effectively distinguish between them based on the ratio of their signals, avoiding wavelengths dominated by hemoglobin or water absorption, which could hinder tissue differentiation.” (ll. 67-72)*

• What role does the printed circuit board (PCB) play in the operation of the handheld fiber-optic tissue sensing device?

*The PCB plays a crucial role in the operation of the handheld fiber-optic tissue sensing device, serving as the central hub for its electronics. Specifically, it houses the driver circuits for the laser diodes and the signal amplification required for the photodiode (ll. 267-268). The PCB ensures that the various components of the device, such as the light sources and detectors, are properly controlled and coordinated. During operation, the Arduino connected to the PCB manages the laser diode drivers, reads and stores the output from the photodiode, classifies the tissue based on the detected signals, and controls the feedback system (ll. 293-296). This compact integration of electronic components through the PCB allows for efficient real-time processing and feedback, which is essential for providing immediate guidance during surgery.*

• How does the microcontroller process the signals from the photodiode to provide real-time feedback to surgeons?

*We appreciate the reviewer’s interest in how our device provides real-time feedback through signal processing. The photodiode current is first converted to a voltage using a transimpedance amplifier, as described in our manuscript. This voltage signal is then digitized by an external analog-to-digital converter, which communicates with the Arduino microcontroller via I2C communication (ll. 305-307). The Arduino retrieves the digital values and collects them over a 200 ms window to ensure sufficient sampling. Subsequently, the data from both wavelengths is averaged to calculate the reflection ratio. This ratio is used to determine the type of bone tissue, allowing the device to deliver accurate, real-time feedback to assist surgeons in differentiating between cancellous and cortical bone (ll. 349-352). We hope this explanation clarifies the role of the microcontroller in our tissue-sensing device.*

• What are the implications of using low-cost and readily available components in the design of the fiber-optic sensing device for widespread clinical adoption?

*We thank the reviewer for highlighting the importance of using low-cost and readily available components in our device. As noted in the manuscript, these components help significantly lower production cost and time. By utilizing affordable and easily accessible materials, our design makes advanced tissue sensing technology more widely available, especially in resource-limited settings or smaller healthcare facilities where more expensive systems, such as robotic-assisted navigation, may not be feasible. Our manuscript states: “Through this novel design, we aim to democratize the availability of tissue sensing technology, making it accessible to a wider range of healthcare practitioners and facilities.” (ll. 50-52)*

• In what ways does real-time audio feedback improve surgical accuracy and outcomes during spinal fusion procedures?

*We appreciate the reviewer’s interest in the role of real-time audio feedback in improving surgical accuracy during spinal fusion procedures. Integrating this feedback mechanism allows surgeons to gain immediate insights into the tissue's optical properties, such as its structure and composition, without having to divert their attention from the surgical field. The audio feedback informs the surgeon in real-time about the proximity to critical anatomical features, such as the cortical border, helping to prevent breaches during pedicle screw placement and enhancing overall accuracy. This improvement in workflow efficiency and precision can lead to better surgical outcomes and reduce the likelihood of complications. We have revised the manuscript to emphasize this point: "The device generates real-time audio feedback to help surgeons prevent pedicle screw breaches and improve overall accuracy during spinal fusion. " (ll. 427-428)*

• What are the limitations of the prototype device, and how might future iterations address these issues?

*We acknowledge that the prototype has certain limitations, primarily concerning its insufficient coupling efficiency, which is lower than anticipated. As stated in our manuscript, the coupling efficiencies were 14% for the 940 nm laser diode and 0% for both the 1310 nm laser diode and the photodiode (ll. 393-398). To address this issue in future iterations, we plan to adopt more reliable assembly techniques for optical coupling. As outlined in the revised manuscript: “To mitigate these shortcomings, reliable assembly tools and processes for butt-coupling have to be established to align the fiber and diode surfaces with high precision.” (ll. 443-444)*

*Additionally, we recognize that further validation of the device on real tissue samples is necessary to confirm the applicability of our findings beyond phantom models (ll. 522-531). The mechanical robustness of the prototype will also need to be assessed in future iterations, particularly to withstand the mechanical forces exerted during spinal surgery (ll. 545-550). This will ensure that the device is both functional and durable in clinical use.*

• How does the device's compact design influence its usability in various healthcare settings?

*We agree with the reviewer that the compact design of our device plays a critical role in enhancing its usability across a variety of healthcare settings. As highlighted in our manuscript, current DRS systems are often bulky and expensive, limiting their adoption, particularly in low-resource settings (ll. 14-16). In contrast, our handheld device offers two important use cases:*

1. *Standalone Use in Low-Resource Settings: The compact size and low cost of our device make it suitable for healthcare facilities with limited access to expensive surgical technologies, such as computer-assisted navigation or robotic systems. Its portability and ease of use could democratize the application of advanced tissue-sensing technology in these environments.*
2. *Add-On for Advanced Surgical Systems: In more resource-rich environments, our device can serve as an add-on to existing computer-assisted navigation or robotic-assisted systems in spinal fusion surgeries. Its integration could enhance the accuracy of pedicle screw placement without requiring significant changes to current surgical workflows.*

*We have revised the manuscript to reflect these aspects: “Our handheld fiber-optic tissue sensing device utilizes DRS to distinguish bone tissues, demonstrating potential to provide surgical guidance in spinal fusion procedures. Thanks to its compact size and low cost, the device is well-suited for healthcare facilities with limited access to expensive surgical technologies, while also being compatible as an add-on to existing computer-assisted navigation or robotic-assisted systems.” (ll. 578-582)*

• What potential improvements could be made to the signal amplification process to enhance the accuracy of tissue differentiation?

*We appreciate the reviewer’s inquiry regarding potential improvements to the signal amplification process. Our current setup offers a maximum amplification of 50 MV/A, while only 6 MV/A is required for accurate tissue differentiation. Given this ample headroom, we believe that the current signal amplification configuration is sufficient and does not require further enhancement to achieve reliable results.*

• How does the device ensure that the feedback provided to surgeons is both timely and reliable during surgery?

*We thank the reviewer for raising this point regarding the timeliness and reliability of the feedback. The PWM frequency used to operate the laser diodes is synchronized with the ADC readout, ensuring simultaneous activation and readout of each laser diode, which facilitates timely signal collection. To accommodate human auditory response times, the readout signals are collected and averaged over a 200 ms window, providing 5 Hz real-time feedback to the surgeon (ll. 346-352).*

*Regarding the reliability of the signal, we agree with the reviewer that it is critical. As emphasized in our manuscript, “Ensuring uniformity in threshold ratio values across prototypes and procedures is crucial to avoid the need for recurrent recalibration by manufacturers or surgeons” (ll. 535-537). We believe this method ensures that the feedback provided is both timely and reliable during surgery.*

• The literature should be improved using the below papers
o Wang, Y., Zhai, W., Zhang, H., Cheng, S., & Li, J. (2023). Injectable Polyzwitterionic Lubricant for Complete Prevention of Cardiac Adhesion. Macromolecular Bioscience, 2200554. doi: <https://doi.org/10.1002/mabi.202200554>
o Yao, R., Ge, Z., Wang, D., Shang, N., & Shi, J. (2024). Self-sensing joints for in-situ structural health monitoring of composite pipes: A piezoresistive behavior-based method. Engineering Structures, 308, 118049. doi: <https://doi.org/10.1016/j.engstruct.2024.118049>
o Fu, Y., Chen, X., Song, W., Kuang, J., Wu, W., Yang, X.,... Liao, Y. (2024). Light-Switch Electrochemiluminescence-Driven microfluidic sensor for rapid and sensitive detection of Mpox virus. Chemical Engineering Journal, 498, 154930. doi: <https://doi.org/10.1016/j.cej.2024.154930>
o Zhou, Y., Xie, J., Zhang, X., Wu, W., & Kwong, S. (2024). Energy-Efficient and Interpretable Multisensor Human Activity Recognition via Deep Fused Lasso Net. IEEE Transactions on Emerging Topics in Computational Intelligence, 1-13. doi: 10.1109/TETCI.2024.3430008
o Jiang, Z., Han, X., Zhao, C., Wang, S., & Tang, X. (2022). Recent Advance in Biological Responsive Nanomaterials for Biosensing and Molecular Imaging Application. International Journal of Molecular Sciences , 23(3), 1923. doi: <https://doi.org/10.3390/ijms23031923>

*We appreciate the reviewer’s suggestion to include additional literature to strengthen our manuscript. Upon reviewing the recommended papers, we found that they primarily focus on materials science, microfluidic sensors, and other areas unrelated to our work on handheld fiber-optic devices for spinal surgery. As our research centers on the development of a real-time tissue-sensing tool based on DRS, the suggested papers do not directly align with the scope of our study. Therefore, we have decided not to include these references in the revised manuscript.*

• What are the broader implications of integrating tissue sensing technology into surgical instruments for other types of surgeries beyond spinal procedures?

*We agree with the reviewer that the integration of tissue sensing technology holds potential for a variety of surgeries beyond spinal fusion. While our manuscript specifically focuses on spinal surgery, DRS has been demonstrated as a useful tool in other minimally invasive surgeries where tissue differentiation is critical. For example, DRS has shown promise in tumor detection and margin assessment in areas such as the bladder, bowels, liver, lungs, breast, and oral cavity (disc 28-40). These broad applications underscore the versatility of DRS across different surgical contexts. We have updated our manuscript to reflect this broader potential in the discussion section, emphasizing DRS's relevance to various types of surgeries: “While our prototype specifically focuses on spine surgery, DRS has been demonstrated as a useful tool in other body regions like the colon, lungs, or breast [44-47].” (ll. 452-453)*

• How does the study address the need for improved surgical accuracy in the context of rising demand for interventions related to age-related degenerative diseases?

*With the increasing prevalence of age-related degenerative diseases, there is a corresponding rise in demand for surgical interventions such as spinal fusion. As these procedures become more common, ensuring speed and accuracy is crucial to avoid complications, revision surgeries, and to improve patient outcomes. We acknowledge this important context in our revised introduction: “Despite the emphasis on achieving surgical accuracy to avoid complications and revision surgeries, accurate screw placement remains a challenge due to the complex anatomy of the spine and the reliance on tactile feedback and anatomical expertise [4, 5].” (ll. 7-10)*

• What future research directions could be pursued to further develop and optimize fiber-optic sensing technologies in surgical applications?

*We appreciate the reviewer’s insightful question regarding future research directions for fiber-optic sensing technologies. One promising area is multi-directional sensing for 3D tissue feedback. Additionally, integrating machine learning algorithms with fiber-optic data could enhance the real-time classification of tissue types during surgery. Another exciting research direction is the combination of DRS with other complementary modalities, which could expand the range of applications beyond spinal surgery. We have reflected this in the revised manuscript: “To further advance fiber-optic tissue sensing, several research directions could be explored. One potential improvement is multi-directional sensing, which would enable directional tissue feedback to detect breaches impending from various angles [56]. Another promising avenue is the integration of machine learning algorithms for real-time classification of tissue types, potentially increasing the speed and reliability of intraoperative feedback [57, 58]. Additionally, combining DRS with complementary sensing modalities, such as ultrasound or electrical impedance, could provide a more comprehensive assessment of tissue properties, broadening the applicability of fiber-optic sensing across various surgical specialties.” (ll. 562-569)*

**Reviewer #3:**

This manuscript presents an interesting prototype—a handheld fiber-optic sensor for differentiating between bone and tissue. However, the authors did not compare the performance of the proposed prototype with conventional fiber-optic sensors. Additionally, the authors did not discuss the pros and cons of this technology compared to other modalities, such as ultrasonography, which is also portable and provides real-time imaging.

*We sincerely thank the reviewer for their insightful comments. While we agree that a direct comparison with conventional fiber-optic sensors and other modalities like ultrasonography would add further value, the primary focus of our paper is on presenting a novel design concept with initial validation results, rather than benchmarking it against existing technologies. Our sensor is a first-of-its-kind device specifically designed for intraoperative bone tissue differentiation in spine surgery, which distinguishes it from conventional fiber-optic sensors as well as ultrasonography. Although ultrasonography provides imaging benefits, it typically requires a learning curve, whereas our device offers a simpler, cost-effective, handheld solution with real-time tissue feedback that does not rely on user interpretation of images. We have added a paragraph on ultrasonography to our revised manuscript: “Intra-operative ultrasonography is cost-effective and provides real-time imaging within a portable device. However, ultrasound has limitations in visualizing bony structures due to poor penetration through dense tissue and low signal-to-noise ratio (SNR), and its efficacy still largely depends on the operator’s expertise [11,12]. These limitations underscore the need for alternative approaches that offer more direct, consistent, and interpretable feedback during spine surgery.” (ll. 17-21)*

*Moreover, our device builds on the success of DRS, a technology that has been benchmarked against Magnetic Resonance Imaging (MRI) for quantifying vertebral bone fat fraction, demonstrating its potential for providing reliable and clinically relevant tissue feedback. DRS has been validated for the detection of cortical breaches in ex-vivo human tissue and in-vivo swine experiments. As we continue to develop this technology, we aim to conduct comparative studies against conventional technologies for benchmarking in future iterations. We have now acknowledged this limitation in the manuscript: “Additionally, in the future, our device will need to be validated against conventional guidance technologies in spine surgery, such as fluoroscopy and computer-assisted navigation. Notably, DRS has previously been benchmarked against Magnetic Resonance Imaging (MRI) for quantifying vertebral bone fat fraction [52], underscoring its potential to provide reliable tissue feedback. Previous work on ex-vivo human tissue and in-vivo swine models has established DRS as a viable tissue sensing technology for spine surgery [16, 20]. Comparative studies will further establish the device’s performance and its potential for integration into clinical practice.” (ll. 538-544)*

The experiments are too simple, and the data is not robust enough to demonstrate the novelty of this work.

*We understand the reviewer’s concern regarding the simplicity of the experiments. We acknowledge that the current experiments are foundational; however, given that this is a technical proof-of-concept, our primary goal was to demonstrate the feasibility of the approach and validate the design. As this is an early-stage prototype, more comprehensive testing, including ex-vivo and clinical trials, will be necessary to further validate the performance. We have clarified in the manuscript that this work presents initial validation and highlights the future steps required for clinical adoption: “We acknowledge that the current experiments are foundational and relatively simple, thus unable to fully assess the performance of our device in a clinical setting. However, as this work represents an early-stage proof-of-concept, the primary goal was to demonstrate the feasibility of this novel approach and validate our design through initial testing. Future optimization will involve more comprehensive evaluations, including ex-vivo trials, validation against conventional guidance technologies, and design iterations to fully validate its potential for clinical translation.” (ll. 515-521)*

Furthermore, Figures 2 and 3 are missing.

*We apologize for any oversight regarding Figures 2 and 3. These figures were included in our submitted version, and we respectfully request the editor to ensure they are visible in the revised manuscript.*

The proposed concept is clinically useful. The reviewer suggests resubmitting the manuscript after adding more experiments (at least ex vivo).

*We appreciate the reviewer’s suggestion to include ex vivo experiments. For these initial experiments, we deliberately opted for bone-mimicking phantom models to control environmental variables and focus on validating the technical performance of the device. Phantoms can be manufactured to match the optical properties of cancellous and cortical bone with high precision, making the results from these studies highly reliable at this early stage. We have added this clarification in the manuscript: “Next, the probe was placed in cancellous bone-mimicking phantom (pure coconut milk with a fat content of 17% [23], providing a highly controlled environment, allowing us to isolate and validate the technical performance of the device without the biological variability inherent in ex-vivo or in-vivo tissue.” (ll. 415-418) Future studies will incorporate both ex-vivo and in-vivo models to fully establish the clinical potential of our device.*

*We sincerely thank the reviewer for their constructive feedback and hope they will consider the technical contributions of this work as a foundation for future experimental validation.*
